# Laboratory studies on the oviposition stimuli of *Culicoides stellifer* (Diptera: Ceratopogonidae), a suspected vector of Orbiviruses in the United States

**DOI:** 10.1186/s13071-018-2891-8

**Published:** 2018-05-16

**Authors:** Dinesh Erram, Nathan Burkett-Cadena

**Affiliations:** Florida Medical Entomology Laboratory, University of Florida, IFAS, 200 9th St. SE, Vero Beach, FL 32962 USA

**Keywords:** *Culicoides stellifer*, Biting midges, Oviposition, Hemorrhagic disease, Ruminants

## Abstract

**Background:**

Biting midges of the genus *Culicoides* (Diptera: Ceratopogonidae) exert a significant impact on animal agriculture worldwide because they transmit bluetongue virus (BTV) and epizootic hemorrhagic disease virus (EHDV) to ruminants. Without effective vaccines, BTV/EHDV vector management strategies are needed, particularly in commercial white-tailed deer (WTD) facilities. However, detailed information on the ecology of midge immatures in/around cervid operations is currently lacking. Towards filling this knowledge gap, we conducted two-choice oviposition experiments with field-collected *Culicoides stellifer* Coquillett (a suspected vector of BTV/EHDV in the USA) under laboratory conditions to examine which natural source from the larval habitat is relatively more attractive for midge oviposition.

**Methods:**

Field-collected *C. stellifer* females (CDC-UV light traps) were given a blood meal from live chicken and examined for their oviposition preferences for individual (or mixed) potential larval habitat oviposition stimuli in two-choice bioassays. Substrates included mud from *C. stellifer* habitat, mud from allopatric site, vegetation (*Sphagnum* spp. mosses), field water, WTD manure and de-ionized water (control).

**Results:**

The majority of midges (91%) oviposited in only one dish, with few females (9%) ovipositing in both the dishes. Gravid females demonstrated an overall oviposition preference for substrates with mud and vegetation from the larval habitat, depositing a significantly higher proportion of eggs on mud (52.3%) and vegetation (81.8%) than on controls (≤ 18.2%) (*P* ≤ 0.0320). Moreover, greater number of eggs per female were deposited on mud (29.5–40.7 depending on trial) and vegetation (38.2) than on controls (≤ 5.8). WTD manure, field water and mud from allopatric site were not found to be more attractive than controls for oviposition. Combining individual substrates (mud + WTD manure; mud + moss + WTD manure + field water) did not elicit greater oviposition responses than mud or moss alone.

**Conclusions:**

Management strategies to discourage *C. stellifer* oviposition in/around commercial cervid facilities should likely focus on mud and/or vegetation, rather than WTD manure. However, further studies are needed to examine whether the spatial distributions of *C. stellifer* and *Sphagnum* spp. moss are correlated, and to determine whether targeting vegetation in/around cervid facilities can contribute to reductions in local midge densities.

**Electronic supplementary material:**

The online version of this article (10.1186/s13071-018-2891-8) contains supplementary material, which is available to authorized users.

## Background

Biting midges in the genus *Culicoides* (Diptera: Ceratopogonidae) are of significant medical and veterinary importance worldwide because the bites of females can result in major annoyance, pathogen transmission, and/or hypersensitivity reaction in susceptible hosts [1, 2]. Among the several pathogen classes that *Culicoides* species transmit, two arboviruses affecting ruminants, bluetongue virus (BTV) and epizootic hemorrhagic disease virus (EHDV) (Genus *Orbivirus*, Family *Reoviridae*), cause significant morbidity, mortality, and economic losses in animal agriculture worldwide [3]. Although these viruses affect a variety of domestic and wild ruminants, BTV affects primarily sheep and cattle, while EHDV affects mainly white-tailed deer (WTD) (*Odocoileus virginianus* Zimmermann) and also cattle [4]. Unfortunately, no effective midge control strategies currently exist, primarily because many of the fundamental biological aspects of the *Culicoides* species associated with BTV/EHDV transmission are poorly understood [1, 2].

Currently, the only confirmed vectors of BTV/EHDV in North America are *Culicoides sonorensis* Wirth & Jones (BTV and EHDV), and *Culicoides insignis* Lutz (BTV) [3, 5–7]. In the USA, *C. sonorensis* has been known to occur primarily through the western states and scattered/rare through the east [2, 8–10]. However, recent findings of this species in southern Ontario, Canada suggest that *C. sonorensis* may have undergone a major range expansion towards the northeast [11], or is poorly documented in some parts of its range. The distribution of *C. insignis* in the USA is primarily limited to Florida and neighboring states; however, recent reports suggest a northward range expansion of this species as well [10].

Entomological investigations during BTV/EHDV outbreaks in several southeastern states (Mississippi, Alabama, Georgia and North Carolina) have found that the confirmed vectors *C. sonorensis* and *C. insignis* were either absent or only present in very low numbers in light trap collections or animal aspirations [8, 9, 12–14]. In addition, the distributions of *C. sonorensis* and *C. insignis* overlap only partially with that of EHDV in the USA [15]. Moreover, field associations between these confirmed vectors and BTV/EHDV outbreaks in WTD have been rare, with only one report from Kentucky documenting *C. sonorensis* to be the predominant midge species during disease outbreaks [6]. The lack of temporal and spatial association of BTV/EHDV outbreaks with *C. sonorensis* or *C. insignis* abundance in several states suggests that other *Culicoides* species are likely involved in virus transmission, particularly in the southeastern USA. Two biting midge species that have been suggested as putative vectors of BTV/EHDV in these states are *Culicoides debilipalpis* Lutz and *Culicoides stellifer* Coquillett.

*Culicoides stellifer* is known to occur throughout most of the USA (between latitudes approximately 49°N to 25°N) with its distribution in North America ranging from Montana and Nova Scotia in the North to California and Florida in the south [16]. This midge species is one of the most common species found in the eastern USA, and its immature stages have been suggested to occur in a variety of organically enriched mud substrates [16]. However, other than general descriptive reports of where the larvae were previously found, studies characterizing the larval habitat of *C. stellifer* are lacking, which has significant implications limiting the design and implementation of management/survey strategies against this putative vector species. Much of our knowledge on the larval habitat characteristics of BTV/EHDV vectors in North America arises from studies on *C. sonorensis*, the larvae of which are known to occur mainly in animal-waste enhanced muds [17–23]. Comparatively, little detailed information is available regarding the habitat characteristics of other suspected/potential vector *Culicoides* species associated with BTV/EHDV transmission in North America. Moreover, oviposition in *Culicoides* species worldwide has received very little attention to date [18, 24–27], with the oviposition of North American *Culicoides* species being virtually unexplored [25, 27]. Therefore, many of the parameters involved in the oviposition site selection of *Culicoides* species pertinent to animal virus transmission in North America and other parts of the world are unknown. In mosquitoes and other dipterans, many physical and/or chemical cues have been shown to influence insect oviposition [28–32]. Similar studies on *Culicoides* species are lacking and generating such fundamental information may provide clues towards colonization of target species and eventual establishment of control strategies by targeting gravid females and/or immature stages [33, 34]. Thus, with a long-term goal of developing midge management strategies, particularly in commercial cervid facilities, we conducted two-choice oviposition experiments on field-collected *C. stellifer* midges under laboratory conditions to examine which natural substrates from the larval habitat (mud, vegetation, standing water, or host animal manure) are relatively more attractive for midge oviposition.

## Methods

### Live biting midge collection

**Fig. 1:**
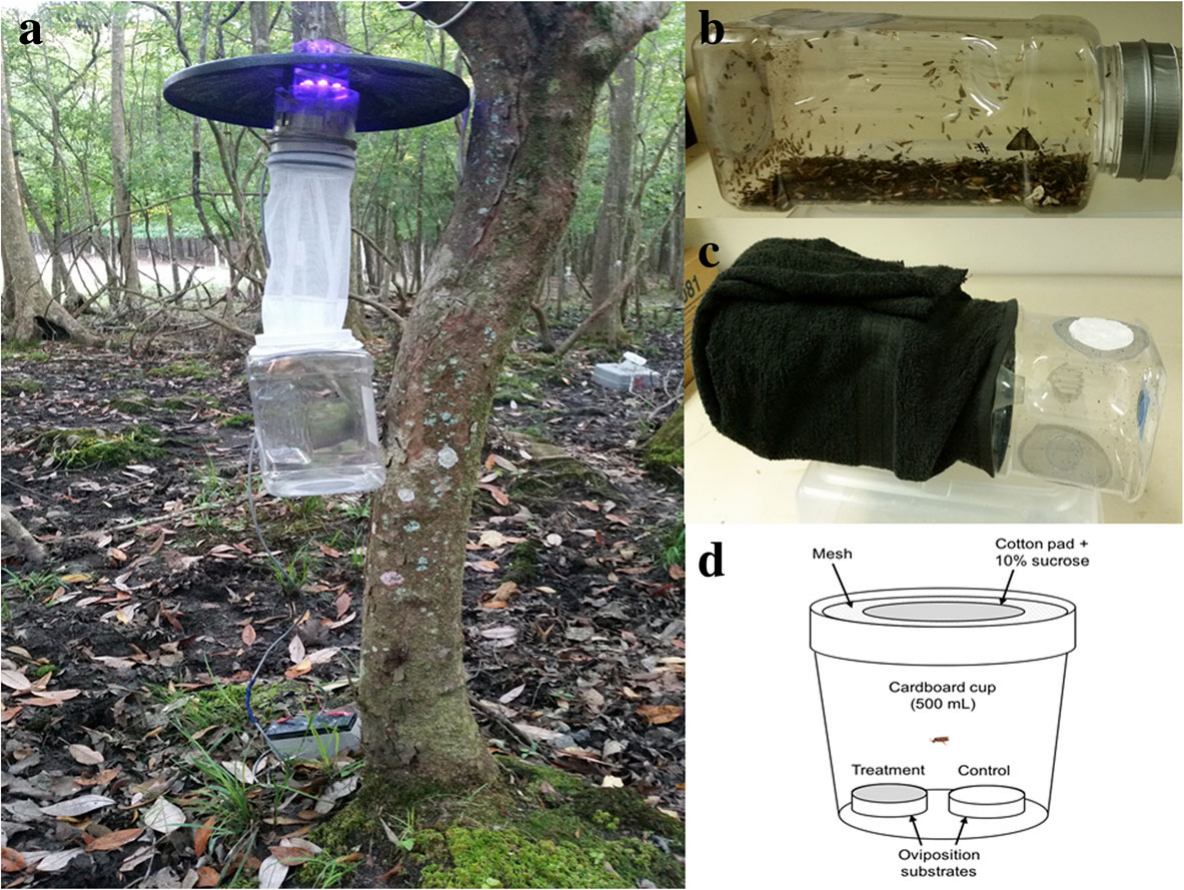
CDC miniature light traps set up overnight on the larval habitat of *C. stellifer* at a commercial deer farm in Quincy, FL, USA, to capture live midges (**a**). Trap collections returned to laboratory the next morning (**b**). The trap collection chamber connected to a sorting chamber and covered with a black cloth for *Culicoides* species to move into the sorting chamber (**c**). Set-up of the two-choice oviposition bioassays (**d**)

Live biting midges were collected from a private game ranch in Quincy, Gadsden County, FL, USA, (GPS coordinates: 30°28'35.2"N, 84°38'54.9"W) using CDC miniature UV light traps connected to live midge collection chambers. The CDC light traps (model #2836BQ, BioQuip products, Rancho Dominguez, CA, USA) utilized blacklight LED arrays as the sole attractant (catalog #2790V390, BioQuip products, Rancho Dominguez, CA, USA). The live midge collection chamber consisted of 1.9 or 3.8 l plastic canisters (Mainstays, Walmart, Vero Beach, FL, USA) with a circular hole (10 cm diameter) cut through the lid allowing the passage and connection of a standard drawstring sleeve (Fig. 1a). An 8 cm diameter hole (or pair of holes) were drilled through the canister walls and sealed with a fine mesh to allow the escape of air flowing from the fan of the light trap blowing into the canister but also prevent insect escape. To separate live *Culicoides* from the substantial by-catch (Fig. 1b), the trap collection chamber was returned to the laboratory and connected to a sorting chamber (Fig. 1c). The sorting chamber consisted of a similarly sized container connected to the collection chamber at their lids (using duct tape) and separated by a screen (20-mesh size) and a funnel (diameter of the narrow end ~2.0 cm). The collection chamber was then wrapped with a black towel to create a darker environment so that insects displaying positive phototactic behavior in the collection chamber would voluntarily migrate to the brighter (sorting) chamber. The sorting chamber was also provided with cotton pads moistened with 10% sucrose solution as additional incentive. The mesh screen between the two chambers excludes the passage of larger insects. *Culicoides* females sorted in this manner were transferred to 500 ml non-waxed cardboard cups sealed with no-see-um netting on top using a mouth aspirator. *Culicoides stellifer* females were sorted from other *Culicoides* spp. using morphological characters [16] of anesthetized midges under a stereoscopic microscope (Nikon SMZ 745). Biting midges were anesthetized using one drop of triethylamine (TEA) (Fisher Scientific 04884-100, Atlanta, GA, USA) diluted 10× in ethanol pipetted onto a piece of cotton that was placed on the mesh of paper cup and covered with a Petri dish [35]. TEA exposure periods were 2–3 min or until all midges stopped moving. *Culicoides stellifer* females were transferred to paper cups as above and provided with cotton pads moistened with 10% sucrose solution, changed daily, until use.

### Oviposition substrates

Types of oviposition substrates selected were based on personal observations and published reports [23, 26, 27]. Field water was collected into 50 ml conical tubes by dipping the tube into shaded puddles in lowland hardwood forests in Quincy, FL, USA, at the approximate location of *C. stellifer* light trap collections. Year-round sampling from this location indicated that *C. stellifer* constituted > 75% of light trap and emergence trap collections (data not shown). White-tailed deer manure (< 6 h old) [23, 27] was collected from the floor of breeding pens from a commercial WTD facility in Okeechobee, FL, USA. Diet of the deer included a locally prepared commercial feed of 16% deer pellets (Odolicious 200, Walpole Feed and Supply Co., Okeechobee, FL, USA), corn treats, peanut hay, and browse consisting of wild grape vines, oak leaves and grass. No animals were treated with insecticides or antibiotics for at least one month prior to the days of manure collection. Moss (*Sphagnum* spp.) [26] was sampled from the shaded forest floor in Quincy, FL, USA, in the vicinity of verified larval development sites using a trowel. ‘Sympatric mud’ was sampled from the same lowland forest location as field water and moss, also using a trowel. ‘Allopatric mud’ was sampled from MacArthur Agro-ecology Research Center, Lake Placid, Highlands County, FL, USA (GPS coordinates: 27°9'16.4"N, 81°11'51.7"W). Sampling at this location indicated that *C. stellifer* was a very minor constituent of the biting midge community (< 1% of *Culicoides* in light traps and no *C. stellifer* emerged from this mud but mainly *C. insignis*) (data not shown).

Oviposition substrates were presented to blood-fed midges in plastic Petri dishes (35 × 10 mm) with 1 g of the chosen substrate and 1.5 ml of deionized water (DI control). Mud, moss or WTD manure (each homogenized individually using mortar and pestle) were placed in the dish and covered with cotton and filter paper. Field water substrate consisted of cotton and filter paper moistened with 1.5 ml of field water. Control substrates consisted of cotton and filter paper. Moisture levels across control and treatment dishes were kept constant by adding 1.5 ml deionized water to each dish. The ‘combined’ substrates were prepared by mixing equal parts (by weight) of sympatric mud, moss, and WTD manure and 1.5 ml of field water. The substrates with sympatric mud + WTD manure were prepared by mixing 3 parts mud and 1 part manure. Each Petri dish was hot-glued to the bottom of the paper cup to minimize Petri dish movements within the cup that may crush females during handling.

### Oviposition assays

*Culicoides stellifer* females were fed upon the blood of a live, restrained chicken after a starvation period of 6–24 h. Starved females were first transferred to 50 ml conical tubes (in groups of 20–30), and the conical tubes, fitted with fine mesh screen caps, were pressed downwards against the breast of the chicken for 30–45 min. Subsequently, blood-fed females were sorted from unfed females (partially blood-fed and fully-engorged females not discriminated) using TEA anesthesia as described above and placed in separate paper cups for 48 h to allow egg development. Cotton pads moistened with 10% sucrose solution were replaced daily. Thereafter, individual females were transferred to 500 ml paper cups in which two oviposition substrates were previously placed, and the females were monitored and allowed to oviposit for 14 days (Fig. 1d). At the end of the two weeks or following midge death, eggs on the oviposition substrates were counted and midges were dissected to determine the number of retained eggs, if any. Cotton pads moistened with 10% sucrose solution given to the females were replaced daily.

Altogether eight different two-choice experiments were conducted testing the relative importance of various environmental substrates on midge oviposition (Table 1). Four experiments tested a single substrate (sympatric mud or moss or field water or WTD manure) *vs* a control (DI water); one experiment tested combined substrates (mud + moss + WTD manure + field water) *vs* a DI water control; two experiments tested substrate against other substrates (sympatric mud + WTD manure *vs* sympatric mud control, and sympatric mud *vs* allopatric mud); and one experiment tested control *vs* control (DI water). Each experiment had at least 5 replicates using a single midge per replicate and was repeated at least two times (Table 1). Environmental conditions in the walk-in incubator where the experiments were conducted were 26 ± 1 °C, 60–80% RH and 16:8 (L:D) h photoperiod cycle.

**Table 1:** Summary of the oviposition experiments on *C. stellifer* under different two-choice conditions

Experiment	Treatment *vs* control	Trials^a^ (replicates within each trial)	Blood-fed females (*n*)	Gravid females (*n*)	Oviposited females (*n*)	Odds ratio (95% CI)	*P*-value^b^
1	DI control *vs* DI control	2 (6)	12	10	3	1.0 (0.2–4.1)	0.9890
2	Field water *vs* DI control	2 (6)	12	9	2	0	1.0000
3	Sympatric mud *vs* DI control	2 (6)	12	11	6	4.4 (1.7–11.8)	**0.0042**
4	WTD manure *vs* DI control	2 (7)	14	10	4	0.4 (0.1–2.0)	0.2500
5	Moss *vs* DI control	2 (8–9)	17	11	11	9.3 (1.5–59.0)	**0.0320**
6	Combined substrates *vs* DI control	3 (5–6)	17	13	5	6.6 (2.3–19.2)	**0.0014**
7	Sympatric mud + WTD manure *vs* Sympatric mud	5 (5–6)	27	21	7	0.67 (0.2–2.6)	0.5700
8	Sympatric mud *vs* Allopatric mud	2 (6–8)	14	13	6	6.2 (2.2–19.2)	**0.0007**

^a^Trials within an experiment were not significantly different (*P* > 0.05)

^b^Significant *P-*values (odds ratio) shown in bold (*P* < 0.05)

*Abbreviations*: *WTD* white-tailed deer, *CI* confidence interval

### Statistical analysis

Only gravid females were considered for statistical analyses and non-gravid females were excluded from the analyses. Variation in the gravid levels (number of eggs developed within individual midges) of field-collected females collected across the experiments was analyzed using generalized linear models (GLM) under a negative binomial distribution with log link function. The proportion of egg batch deposited by individual females across experiments was analyzed using GLM with binomial distribution of the residuals. The preferences of females in the two choice experiments (treatment *vs* control) were analyzed using generalized estimation equations (GEE) to estimate the odds of a female choosing the treatment substrates over controls, in addition to examining differences between trials. The GEE models were fitted with a binomial distribution incorporating a logit link function taking into consideration the proportion of eggs retained by females that deposited eggs. As each midge was given two choices for oviposition (treatment *vs* control), data obtained from the two substrates were related; therefore, each midge within a given trial was considered a cluster and the models were fitted assuming an exchangeable working correlation structure [36, 37]. Differences in the proportion of females ovipositing on one or both the dishes was analyzed using a t-test. Statistical analyses were conducted using R statistical software version 3.30-3 [38] with the packages *MASS* [39], *car* [40] and *geepack* [41] or SAS version 9.4 (PROC TTEST) [42].

## Results

**Fig. 2:**
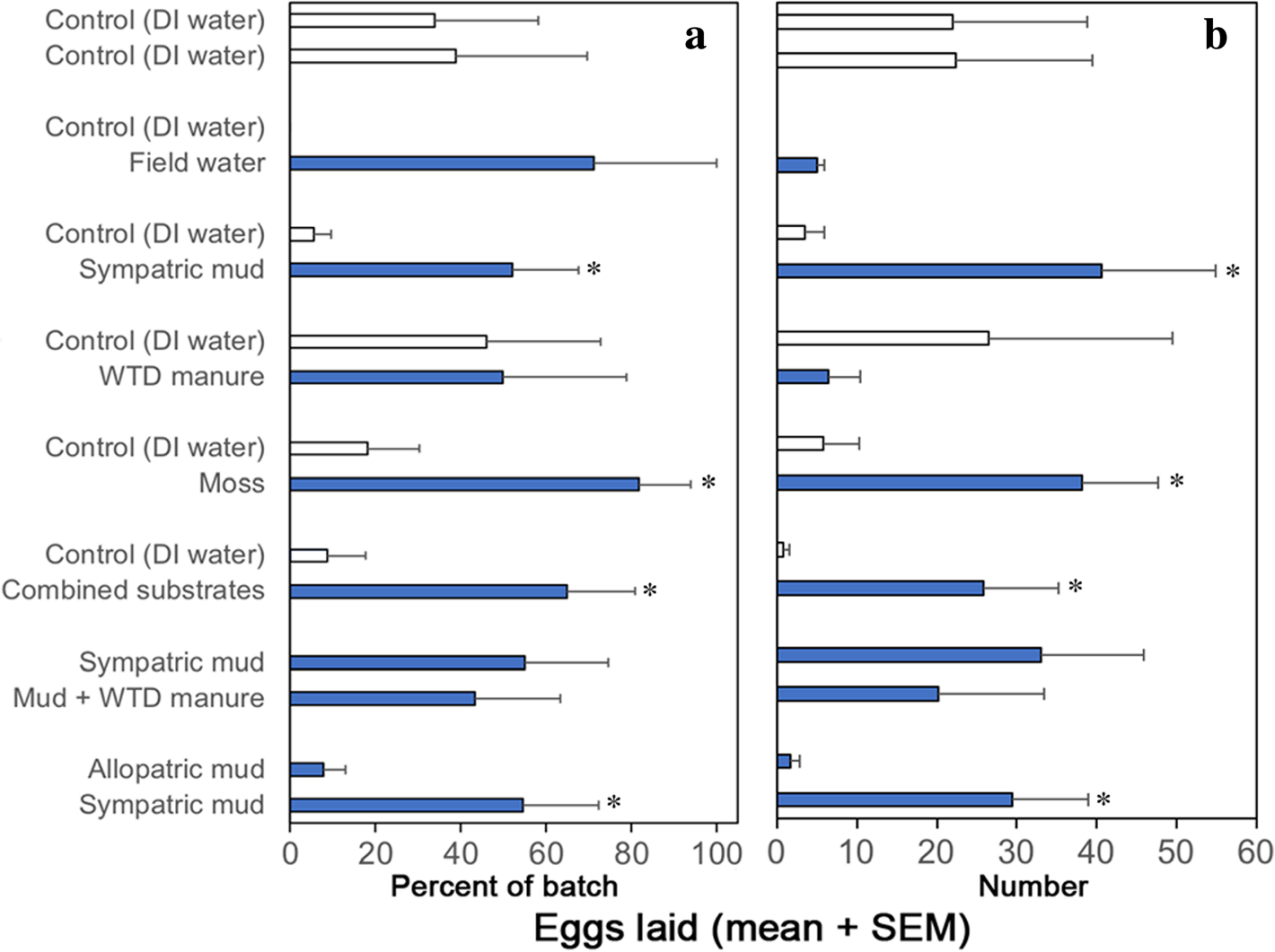
Oviposition preferences of *C. stellifer* under different two-choice conditions. The percentage of egg batch deposited (**a**), and the number of eggs deposited (**b**), by individual females across all experiments. Asterisk indicates that midge preference for treatment substrates over controls within each experiment (odds ratio) was significant (*P* < 0.05). White bars indicate control (DI water)

Overall, the proportion of females that developed an egg batch following a blood meal (of the total females collected) was high and ranged from 65% (11/17 females during moss *vs* DI control trials) to 92% (11/12 females during sympatric mud *vs* DI control trials) across the study (Table 1). The average size of egg batch was 35.9 ± 2.5 (mean ± SE) eggs per female; however, the number of eggs developed within individual females (gravid levels) varied significantly, from a low of just 4 eggs, to a high of 119 eggs (LR *χ*^2^_7, 97_ = 30.5, *P* < 0.0001). Moreover, not all gravid females deposited eggs; the proportion of gravid females that successfully deposited eggs varied greatly from 22% (2/9 gravid females during field water *vs* DI control trials) to 100% (11/11 gravid females during moss *vs* DI control trials) (Table 1). Furthermore, the proportion of egg batch deposited by individual females (treatment + control) varied significantly across experiments from 58% (sympatric mud *vs* DI control trials) to 100% (moss *vs* DI control trials) (LR *χ*^2^_7, 97_ = 916, *P* < 0.0001) (Fig. 2a, b).

During sympatric mud *vs* DI control trials, midges deposited a higher proportion of eggs on mud substrates (52.3 ± 15.4%) (mean ± SE) than on DI controls (5.6 ± 4.2%). The odds of a female choosing the mud substrate for oviposition was 4.4 times higher than DI controls (odds ratio [OR] = 4.4; 95% CI: 1.7–11.8; *P* = 0.0042), with no significant difference between the two trials conducted (Wald *χ*^2^_1, 10_ = 0.02, *P* = 0.8890) (Fig. 2a, b, Table 1). During moss *vs* DI control trials, midges deposited a higher proportion of eggs on moss substrates (81.8 ± 12.2%) than on DI controls (18.2 ± 12.2%). The odds of a female choosing the moss substrate was 9.3 times higher than DI controls (OR = 9.3; 95% CI: 1.5–59; *P* = 0.0320) and the two trials conducted were not significantly different from each other (Wald *χ*^2^_1, 10_ = 0.30, *P* = 0.5860). During field water *vs* DI control trials, the proportion of eggs deposited on field water treatment (71.4 ± 28.6%) was much higher than DI controls (0.0 ± 0.0); however, the odds of a female choosing the field water substrate for oviposition over DI controls were very low (OR = 0.0; 95% CI: 0.0–0.0; *P* = 1.0000), with no significant difference between the two trials conducted (Wald *χ*^2^_1, 8_ = 0.08, *P* = 0.7700). During WTD manure *vs* DI control trials, the proportion of eggs deposited on WTD manure (50 ± 28.9%) was slightly higher than on DI controls (46.2 ± 26.8), but the odds of a female choosing WTD manure over DI controls were low (OR = 0.4; 95% CI: 0.1–2; *P* = 0.2500) and the two trials conducted were not significantly different from each other (Wald *χ*^2^_1, 9_ = 0.09, *P* = 0.7400) (Fig. 2a, b, Table 1).

During combined substrates *vs* DI control trials, midges deposited a higher proportion of eggs on combined substrates (65 ± 15.9%) than on DI controls (8.9 ± 8.9%), with the odds of a female choosing the combined substrates being 6.6 times higher than DI control substrates (OR = 6.6; 95% CI: 2.3–19.2; *P* = 0.0014); the three trials conducted were not significantly different from each other (Wald *χ*^2^_2, 12_ = 0.03, *P* = 0.8620). During sympatric mud + manure *vs* sympatric mud control trials, the proportion of eggs deposited on the mud + manure substrates (43.4 ± 20%) was slightly lower than mud controls (55.1 ± 19.5%), and the odds of a female choosing the mud + manure substrates for oviposition over mud control substrates were low (OR = 0.67; 95% CI: 0.2–2.6; *P* = 0.5700); the five trials conducted were not significantly different from each other (Wald *χ*^2^_4, 20_ = 1.29, *P* = 0.2560). During sympatric mud *vs* allopatric mud trials, midges deposited a higher proportion of eggs on sympatric mud substrates (54.7 ± 17.7%) than on allopatric mud controls (8 ± 5.2%). The odds of a female choosing sympatric mud was 6.2 times higher than allopatric mud (OR = 6.2; 95% CI: 2.2–19.2; *P* = 0.0007) and the two trials were not significantly different from each other (Wald *χ*^2^_1, 12_ = 0.17, *P* = 0.6810). During the double control trials (DI control *vs* DI control), the odds of a female choosing one substrate over the other was low (OR = 1; 95% CI: 0.2–4.1; *P* = 0.9890) (mean proportion of eggs deposited on either substrate ≤ 38.9%) with no significant difference between the two trials conducted (Wald *χ*^2^_1, 9_ = 0.22, *P* = 0.6400) (Fig. 2a, b, Table 1).

**Fig. 3:**
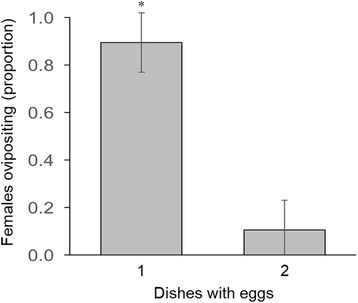
Proportion of females that oviposited on one or both dishes across all experiments. Asterisk indicates significant differences as determined by *t*-test (*P* < 0.05)

A significantly higher proportion of females (91%; 40/44) across the experiments deposited all their eggs on a single dish, compared to females that oviposited in both assay dishes (*t* = 8.43, *df* = 14, *P* < 0.001) (Fig. 3). Throughout the study, only four females deposited eggs in both substrate dishes, each from a different experiment. In two of these, the female laid most of the eggs in one dish (53 eggs in sympatric mud dish *vs* 6 in DI control dish [Additional file 1: Table S3], 75 eggs in mud dish *vs* 3 in mud + WTD manure dish [Table S7]). In the combined substrates *vs* DI control experiment one female laid nearly equivalent numbers of eggs in the two dishes (5 and 4) (Table S6). In DI control *vs* DI control experiment one female laid 11 and 55 eggs in the two control dishes, respectively (Additional file 1: Table S1). All other females (*n* = 40) laid all eggs in just one of the assay dishes (Fig. 3; Additional file 1: Table S1-S8).

## Discussion

Overall, our results suggest that mud and vegetation from the larval habitat provide stronger oviposition cues to *C. stellifer* than field water or host animal manure. However, whether these cues are olfactory or tactile in nature is currently unknown and remains to be examined in further studies. In general, midges deposited a distinctly higher proportion of eggs on substrates with mud or vegetation than on DI controls, but the proportion of eggs deposited on field water or WTD manure substrates was not much different to those deposited on DI controls. We further hypothesized that the individual substrates chosen from the larval habitat may have a synergistic effect on midge oviposition. However, although a higher proportion of eggs were deposited on the combined substrates over DI controls, the total proportion of eggs (treatment + control) deposited during these trials (combined *vs* DI controls) was noticeably lower (73.9%) than those deposited during experiments using moss or WTD manure alone (≥ 96.2%) (*vs* DI controls) (Fig. 2a, b). This suggests that the effect of combined treatment on midge oviposition was sub-additive rather than synergistic, which is possibly due to an inhibitory effect of one or more of the individual sources on *C. stellifer* oviposition. Previously, sand flies (*Lutzomyia longipalpis* Lutz & Neiva and *Phlebotomus papatasi* Scopoli) deposited significantly more number of eggs on substrates containing frass from laboratory colonies; however, the effect of substrates containing a combination of frass and conspecific eggs was found to be sub-additive [43].

Mud from the larval habitat (irrespective of the presence of WTD manure) appears to play a strong role in positively influencing the oviposition of *C. stellifer*. This is evident from the mud + WTD manure *vs* mud control experiments where the mean proportion of eggs deposited on either substrate was not distinctly different. However, the mean proportion of eggs deposited during mud *vs* DI control trials was distinctly higher on mud substrates over DI controls. Moreover, during subsequent experiments, *C. stellifer* females deposited a higher proportion of eggs on mud from a sympatric site (larval habitat) over mud from an allopatric site. Currently, the biotic/abiotic factors in the mud that play key roles in influencing midge oviposition are not known; also, unknown are the reasons for the preference of *C. stellifer* gravid females for the larval habitat mud (sympatric mud) over allopatric mud. However, possible hypotheses include: (i) presence of residues from dead/decaying organic matter such as leaf litter, wooden debris, or manure from certain animals that may be more attractive than others [18, 22, 44–46]; (ii) differences in chemical properties that may play a role in the spatial ecology of midges; for example salinity has been shown to influence oviposition in *C. sonorensis* [25, 47–49]; (iii) variation in diversity of the microbial communities in different muds with certain microbial taxa possibly being more attractive than others [50–53]; (iv) chemical cues from conspecifics, competitors or predators that may attract/repel gravid females [28, 54–57]; or (v) chemical cues from certain types of vegetation that may influence midge oviposition [58–60]; (vi) site fidelity [61–63]; or (vii) some oviposition cues originating from the larval habitat may be species specific [64]. However, further studies will be needed to test these hypotheses in *Culicoides* species.

Commercial cervid farming is a growing industry in the United States with an estimated total economic impact of around $2.3 billion annually [65]. As such, orbiviruses such as BTV/EHDV transmitted by *Culicoides* species pose a major threat to cervid farming industry as they cause hemorrhagic disease in WTD that can result in high mortality in deer populations and significant economic losses to deer farmers [4]. While the immature stages of *C. sonorensis* (a confirmed vector of BTV and EHDV in North America) are typically found in animal-waste enhanced muds, the oviposition preferences of gravid females for muds enhanced with different farm animal manures is unknown to date [27]. We hypothesized that WTD manure acts as a strong oviposition attractant/stimulant for *C. stellifer*. However, our results indicate that white-tailed deer manure does not have a major impact on midge oviposition. This is evident from the white-tailed deer manure *vs* DI control experiments as well as from mud + white-tailed deer manure *vs* mud control trials, where the proportion of eggs deposited on white-tailed deer manure substrates was not much different to those deposited on controls. Our findings demonstrate that WTD manure does not play a significant role in the oviposition of *C. stellifer* and suggest that the field sites receiving manure influx from WTD particularly in commercial cervid farms may not be relatively more attractive for *C. stellifer* oviposition over muds without WTD manure. This suggests that manure management, which has been proposed as one of the cultural management strategies for filth fly control [66–68] and to some extent for *C. sonorensis* control [20] on livestock operations, may not be an effective strategy to reduce *C. stellifer* populations in/around cervid facilities. However, it is important to note that the site from which sympatric mud samples were collected for this study had a variety of cervid as well as bovid species free ranging across the property with the animals frequently visiting this site. It is currently uncertain whether or to what extent influx of manure residue from other cervids or bovids occurred into the mud that could have potentially influenced midge choices in this study. Moreover, if olfactory cues from animal manure play a bigger role in midge oviposition than contact cues, it may cause potential errors in the recognition of the cue source by midges as the substrates in this study were placed in relatively small sized paper cups. Interestingly, the total proportion of egg batch (treatment + control) deposited by midges during the experiments involving WTD manure was very high (≥ 96.2%; Fig. 2a, b) compared to most of other experiments, which possibly suggests a positive impact of manure odors on oviposition. Therefore, further studies using, for example wind-tunnel Y-choice olfactometers that prevent physical contact of midges with the substrate, may provide a better assessment of the role of animal manure on midge oviposition. Previous studies on certain dung breeding *Culicoides* species [46], house flies [45] and stable flies [44] suggested that some animal manures are more attractive for oviposition than others. Furthermore, age of manure was shown to influence insect oviposition as some insects prefer fresh manure while some others prefer aged manure [69] (but only fresh WTD manure [< 6 h old] was used in this study). Moreover, diet of the animals can also change the attractiveness of resulting manure for insect oviposition [70]. Therefore, these findings should be interpreted cautiously as the role of any of these factors on the oviposition of *C. stellifer* or other related species is unknown and further studies will be needed to examine whether the findings of this study are biologically significant.

Vegetation (*Sphagnum* spp. mosses) from the larval habitat appears to be a key factor in influencing the oviposition of *C. stellifer*. This is evident from the moss *vs* DI control trials where a distinctly higher mean number/proportion of eggs were deposited on moss substrates over DI controls (Fig. 2a, b). Another result that stands out in this experiment is that the proportion of egg batch deposited by females was the highest (100%) among all experiments, indicating a strong positive impact of the moss on midge oviposition (Fig. 2a). The effect that moss has on midge behavior/physiology that encourages the females to deposit 100 % of their egg batch compared to the other natural sources examined is currently unknown and remains to be investigated in future studies. Previously, *Sphagnum* spp. moss and other vegetation (*Juncus* spp.) were shown to elicit a strong oviposition response in *Culicoides impunctatus* Goetghebuer, the Scottish biting midge, under laboratory conditions [26], supporting our own findings. Moreover, *C. impunctatus* larval densities in the field were found to be significantly correlated to the distribution of *Sphagnum* spp. and *Juncus* spp. along with a variety of abiotic factors [71, 72]. Interestingly, certain members of the *piliferus* group in North America, such as *Culicoides bickleyi* Wirth & Hubert and *Culicoides piliferus* Root & Hoffman, were previously reared from *Sphagnum* spp. bogs [73, 74], while larvae of numerous other midge species were found in habitats that had *Osmunda* fern bogs or other grasses [16]. Similarly, the distribution of some salt marsh *Culicoides* species was closely associated with vegetation such as *Spartina alterniflora* Loiseleur or *Distichlis spicata* (L.) Greene in North Carolina, USA [75]. Moreover, vegetated water bodies have been often found to harbor larvae of *C. insignis* (a confirmed vector of BTV in the United States) in cattle pastures and other areas [16, 76, 77]. Currently, the role of *Sphagnum* spp. moss and/or other vegetation on the oviposition and ecology of *C. stellifer* and other North American *Culicoides* species in nature is unknown. Whether vegetation has significant roles on the overall reproductive behavior such as swarming, mating, oviposition site selection, oviposition and/or even larval development of biting midges warrants further study. Indeed, a variety of plants have been observed to be used as swarm markers in certain *Culicoides* species [78]. A better understanding of the role of vegetation on the behavior and other life history traits of *Culicoides* species may provide novel insights into the spatial ecology of target midge species and provide clues towards their management by potentially targeting vegetation, particularly in/around livestock facilities. For example, the presence of vegetation and/or floating debris along the shoreline of animal waste lagoons has been shown to increase oviposition of *Culex quinquefasciatus* Say mosquitoes, and removal of this organic matter almost completely eliminated mosquito production from these otherwise productive habitats [79].

Field water, sampled from the larval habitat, appears to not have a major impact on the oviposition of *C. stellifer*. This is evident from the field water *vs* DI control trials where although a high proportion of eggs were deposited on substrates with field water than on DI controls, midge preference for the treatment substrate (field water) over controls (odds ratio) was not significant. This outcome is surprising because the immature stages of ceratopogonids are essentially semi-aquatic and soluble cues in the larval environment are likely present in the open standing water that is in contact with larval development sites. It is possible, however, that our results/interpretations from the field water *vs* DI control experiment is an artifact of the low gravid levels [mean number of eggs deposited 5 ± 1 (SE)] and low proportion of females that oviposited (only 2/9 gravid females deposited eggs) during these trials compared to those during other experiments (Fig. 2a, b, Table 1, Additional file 1: Table S2). Therefore, these results should be interpreted cautiously. Nonetheless, breeding site water was also found to not stimulate oviposition in *C. impunctatus* under no-choice conditions previously [26], generally agreeing with our findings on *C. stellifer*.

The huge variation in oviposition rates among field-collected females in this study was not unexpected. It is possible that variation in the gravid levels, proportion of gravid females (out of total females collected), and females that successfully deposited eggs represents differences in blood meal volumes ingested by midges (partially blood-fed and fully-engorged females were not discriminated in this study) as size of the blood meal can influence insect fecundity [80, 81], or a natural variation in the mated status or age of the field-collected females (older females may have had higher mortality before ovipositing than younger ones). However, variation in the proportion of egg batch deposited by females that did oviposit likely represents a differential likeability of the various substrates for midge oviposition, which can potentially be exploited in future colonization studies of *C. stellifer* (particularly *Sphagnum* spp. moss). Moreover, source of the blood meal can influence insect fecundity [82–84]. Therefore, future studies may also benefit by examining whether a mammalian blood meal (as opposed to avian blood meal used in this study) would cause fecundity variation in *C. stellifer* and other important midge species.

Skip oviposition has been observed in several container-breeding mosquito species where the gravid female distributes its eggs over multiple sites, most likely to reduce larval competition in the resource limited container habitats that could be detrimental to larval survival [85–89]. Although such behavior in *Culicoides* species has never been documented before, our study demonstrates that a minor proportion of *C. stellifer* females do exhibit skip oviposition behavior, as evident from 9% (4/44) of females ovipositing on the two available dishes across the study (Fig. 3). However, in three of these cases, the females deposited most of their egg batch (~90% on average) on one dish and a minor proportion on the second, while one female deposited an almost equal proportion of eggs on both dishes. Although skip oviposition may be advantageous in reducing larval densities in resource limiting habitats such as artificial containers, plant pitchers, or tree holes, the significance of skip oviposition on the life history traits of mud breeding species such as *C. stellifer* is currently unknown. Further studies will be needed to examine the role of skip oviposition on *C. stellifer* ecology, and whether skip oviposition occurs or is more prevalent in tree-hole breeders such as *C. debilipalpis* [16] and in other *Culicoides* species in general.

Insects may use a variety of physical and/or chemical cues that act as attractants or stimulants (or deterrents/repellents) for selecting a suitable oviposition site. Some of the physical cues can be color, optical density, reflectance, texture or temperature [31, 32], while chemical cues can originate from conspecifics, competitors, predators, decomposing organic material, vegetation or microbes [28–30, 55, 90, 91]. Our study suggests a strong role of olfactory and/or tactile cue components on the oviposition site selection of *C. stellifer*. Moreover, visual cues (color) may have also played a role, as the cotton/filter paper placed on the treatment substrates were often discolored through time. Previously, visual cues were also suggested to play a role in the oviposition of certain *Culicoides* species [24, 26]. However, many of the other types of cues potentially utilized by biting midges for oviposition site selection are currently unknown and remain to be identified in further studies.

## Conclusions

Mud and vegetation (*Sphagnum* spp. mosses) from the larval habitat likely provide stronger oviposition cues (olfactory/tactile/visual) to *C. stellifer* than field water or host animal (WTD) manure. Pollution of the mud habitat with WTD manure may not be a critical factor in the oviposition site selection of *C. stellifer*. Thus, management strategies to discourage oviposition of *C. stellifer* midges in/around commercial cervid facilities should likely focus on mud and/or vegetation, rather than WTD manure. Further studies will be needed to identify the key biotic/abiotic factors in the larval habitat mud that influence oviposition site selection in *C. stellifer* and other important species. Moreover, further studies will also be needed to examine whether the spatial distributions of *C. stellifer* and *Sphagnum* spp. moss are correlated, and to determine whether targeting *Sphagnum* spp. moss and/or other types of vegetation in/around cervid facilities can contribute to reductions in local midge densities.

## Additional file

Additional file 1: **Table S1.** Oviposition preferences of *C. stellifer* during ‘DI control *vs* DI control’ trials. **Table S2.** Oviposition preferences of *C. stellifer* during ‘Field water *vs* DI control’ trials. **Table S3.** Oviposition preferences of *C. stellifer* during ‘Sympatric mud *vs* DI control’ trials. **Table S4.** Oviposition preferences of *C. stellifer* during ‘WTD manure *vs* DI control’ trials. **Table S5.** Oviposition preferences of *C. stellifer* during ‘*Sphagnum* moss *vs* DI control’ trials. **Table S6.** Oviposition preferences of *C. stellifer* during ‘Combined substrates *vs* DI control’ trials. **Table S7.** Oviposition preferences of *C. stellifer* during ‘Sympatric mud + WTD manure *vs* Sympatric mud’ trials. **Table S8.** Oviposition preferences of *C. stellifer* during ‘Sympatric mud *vs* Allopatric mud’ trials. (XLSX 26 kb)

## Abbreviations

BTV: Bluetongue virus
CI: Confidence interval
DI: Deionized water
EHDV: Epizootic hemorrhagic disease virus
L:D: Light:dark
RH: Relative humidity
WTD: White-tailed deer
